# Sumac (*Rhus coriaria*) Extract-Loaded Polymeric Nanosheets Efficiently Protect Human Dermal Fibroblasts from Oxidative Stress

**DOI:** 10.1021/acsabm.2c00857

**Published:** 2022-11-25

**Authors:** Melis Emanet, Mayu Okuda, Özlem Şen, Chiara Lavarello, Andrea Petretto, Shinji Takeoka, Gianni Ciofani

**Affiliations:** † Istituto Italiano di Tecnologia, Smart Bio-Interfaces, Viale Rinaldo Piaggio 34, 56025Pontedera, Pisa, Italy; ‡ Waseda University, Waseda Research Institute for Science and Engineering, 3-4-1 Okubo, 169-8555Shinjuku, Tokyo, Japan; § Waseda University, Department of Life Science and Medical Bioscience, 2-2 Wakamatsu, 162-8480Shinjuku, Tokyo, Japan; ∥ IRCCS Istituto Giannina Gaslini, Core Facilities-Clinical Proteomics and Metabolomics, Via Gerolamo Gaslini 5, 16147Genova, Italy

**Keywords:** polymeric nanosheets, sumac (*Rhus coriaria*), natural antioxidants, oxidative stress, human dermal fibroblasts

## Abstract

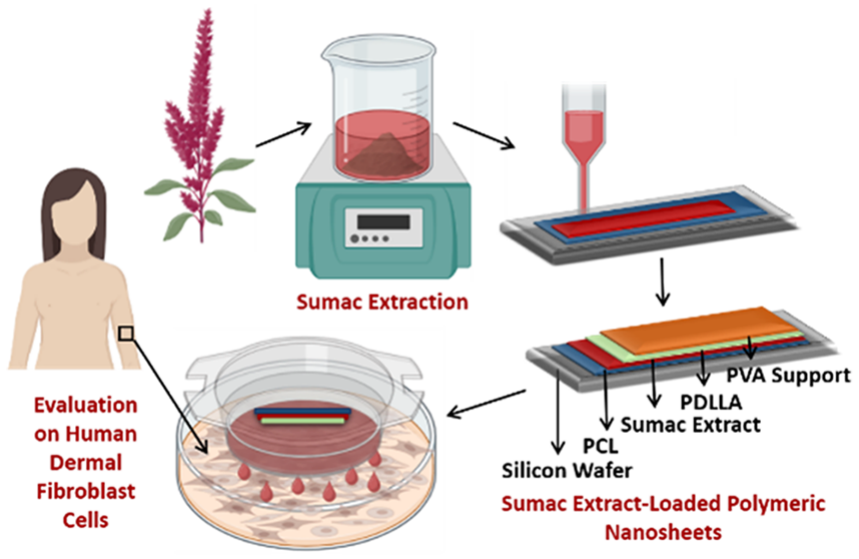

Under healthy physiological conditions, living organisms possess a variety of antioxidant mechanisms to scavenge overproduced reactive oxygen species (ROS). However, under pathological circumstances, endogenous antioxidant systems may not be adequate to eliminate the excessive amount of oxidants, and thus, a continuous exogenous antioxidant income is required. In this regard, sumac (*Rhus coriaria*) extract is a good candidate for therapeutic applications, because of its high content of antioxidant polyphenolic compounds. In this work, sumac extract-loaded nanosheets (sumac-nanosheet) have been exploited for loading and controlled release of sumac extract, envisioning topical drug delivery applications. Sumac extract has been obtained through the solvent extraction method, and polymeric nanosheets have been thereafter prepared through the spin coating-assisted layer-by-layer deposition of polycaprolactone (PCL), sumac extract, and poly(d,l-lactic acid) (PDLLA). The collected data show a rich content of the sumac extract in terms of polyphenolic compounds, as well as its strong antioxidant properties. Moreover, for the first time in the literature, we demonstrated the possibility of efficiently loading such extract in polymeric nanosheets and the suitability of this nanoplatform as a reactive oxygen species scavenger in human dermal fibroblasts treated with a pro-oxidant insult.

## 1 Introduction

Polymeric nanosheets are a novel class of nanostructures with peculiar characteristics in terms of ultrathin thickness and large surface, features that contribute to a uniquely high aspect ratio.^1^ The huge variety of available natural and synthetic polymers that can be used for nanosheet fabrication translates into a wide range of adhesiveness, tunable flexibility, and molecular permeability.^2^ One of the main specific features of the nanosheets is their free-standing ability without the need for supporting material, and this allows an easy assessment of their own physicochemical characteristics.^1^ In addition, their surface can be tailored by using drugs, fluorescent dyes, conductive materials, or even cells in order to broaden the application fields of these structures, especially in the biomedical sector.^2^

Given the specific properties of the nanosheets in terms of polymeric nanoplatforms, the biomedical research is actively focused on their exploitation in wound dressing, tissue engineering, healthcare monitoring, drug delivery applications, and cancer theranostics.^3−5^ Specifically, nanosheets provide obvious advantages in localized drug delivery (such as topical and transdermal) with respect to nanoparticles or oral tablets; moreover, a localized delivery approach overcomes first-pass metabolism and drug side effects, conversely observed in systemic drug delivery.^3^ Despite nanosheets could be particularly suitable for drug delivery applications, their ultrathin structure raised concerns about an efficient drug loading and release profile.^6^ Thus, a great effort has been paid by researchers to improve their drug loading capacity, in order to achieve a suitable level of drug at the targeted area.

“Sandwich” model nanosheets are considered double-sided solid drug delivery formulations and are generally exploited with the aim of drug transfer through topical and transdermal routes.^7^ Going into detail, sandwich model nanosheets are designed with an active compound-loaded layer (reservoir) covered from the two sides with further building blocks, so that both sides of the nanosheet can be separately tuned for tailoring the combined features of the system for a specific application. In topical applications, one side is generally considered as a supporting layer, more resistant to degradation and preventing the release of active molecules; the other side, i.e., the adhesive layer, is more prone to degradation and well-tuned to achieve the desired drug release profile. The adhesiveness of the first layer is a key feature, needed to be tuned depending on the characteristics of the target surface, which could be smooth or wrinkled, as well as dry in topical applications or moist in the case of subdermal routes.^8^ Another important parameter to be considered is the diffusion behavior of the drug molecules from the reservoir at a controllable rate.^8^ According to the desired drug delivery strategy, the parameters affecting the release profile can be tuned by tailoring the used polymers and their concentration, as well as by varying the thickness and the number of layers that are placed in both sides of the reservoir layer of the nanosheet. A good example in this direction is provided by the study of Hatakana et al. In this research, polymeric nanosheets have been obtained through a spin coating of poly(l-lactic acid) (PLA) and poly(lactic-*co*-glycolic acid) (PLGA) building blocks, containing betamethasone valerate (BV) as a model drug.^6^ This nanosheet has been built free-standing thanks to a water-soluble sacrificial layer of poly(vinyl alcohol) (PVA). The polymeric composition of the nanosheet has been tailored in order to tune the BV release according to the desired proposal: since BV is a hydrophobic drug, its loading efficiency increases proportionally to the hydrophobicity of the polymer, which is obtained by higher PLA:PLGA ratios, as well as by the use of high molecular weight PLA.

Sumac is a common name for the genus *Rhus*, and although it comprises 250 individual species, only a few of them have been evaluated for their potential biomedical applications; in particular, *Rhus glabra* (*R. glabra*), grown in North America, is traditionally used in the treatment of bacterial infections, while *Rhus coriaria* (*R. coriaria*), grown in the Canary Islands, Iran, Afghanistan, and southeast of Turkey, is commonly used as a spice and drug, especially for wound healing.^9^ Recently, several studies have been performed on the bioactive ingredients of sumac, representing a rich source of phenolic compounds with strong antimicrobial and antioxidant activity.^10^ Phenols play an important role in plants concerning defense against microorganisms; indeed, they penetrate through the bacterial membrane or the cell wall altering membrane fluidity and cell wall integrity, thus resulting in microorganism death.^11^ Some of the antimicrobial compounds present in sumac are anthocyanins, hydrolyzable tannins, gallic acid, and flavones, in particular myricetin and quercetin.^12^ Concerning production strategies, sumac is able to grow in tropical regions, usually in areas not dedicated to other agricultural products, and this could represent an advantage from an economical point of view: a noncompetitive culture can be carried out aiming at the commercial exploitation of sumac bioactive compounds.^10^

A key factor in the extraction of the bioactive compounds from sumac is represented by the polarity of the exploited solvent, which determines the amount and the species of the compounds themselves. As an example, the literature shows that a hydroethanolic solution (70% v/v), used at 25% w/v on dried sumac, enabled the extraction of about 300 mg/g of phenolic compounds;^12^ conversely, when ethanol was used at 10% w/v, just 15 mg/g of phenolic compounds was extracted.^13^

To the best of our knowledge, just one study in the literature proposes to combine sumac extracts with a nanoplatform, and namely with chitosan nanogels for antimicrobial applications.^14^ Here we present a thorough investigation of the potentialities of sumac extract-loaded polymeric nanosheets, obtained in a sandwich model configuration. The nanosheets were developed through the spin coating-assisted layer-by-layer deposition of poly(caprolactone) (PCL) and poly(d,l-lactic acid) (PDLLA) sheets. Sumac extract was embedded between the PCL and the PDLLA layers, upon oxygen plasma treatment of the first one (to improve PCL hydrophilicity and allow a uniform spreading of the sumac extract hydroalcoholic solution). A PVA layer was used as a sacrificial support layer. Morphology, extract loading amount, and release profile were evaluated, as well as mechanical features in terms of adhesiveness and tensile strength. Biological experiments were carried out on human dermal fibroblasts (HDFs), envisioning dermal application of sumac extract-loaded nanosheets. Biocompatibility and antioxidant activity have been demonstrated through multiple independent assays; eventually, the *in vitro* wound healing capability of the proposed nanoplatform has been verified through a scratch assay.

## 2 Materials and Methods

### 2.1 Extraction of Sumac Bioactive Compounds

Sumac samples (*Rhus coriaria* from southeast Turkey) have been obtained by local producers. In order to maximize the concentration of the extracted active compounds and to develop a reproducible process, samples underwent freeze-drying (16 h after a 24 h treatment at −80 °C) before hydroethanolic extraction.^15^ Briefly, 3 g of freeze-dried sumac powder was dispersed in 40 mL of ethanol aqueous solution (1:1 v/v) and continuously stirred at 180 rev/min on a shaker at room temperature overnight. Thereafter, the extract mixture was filtered by using Whatman grade 1 paper filter for the elimination of solid residues; for further purification, the mixture was centrifuged three times at 8000*g* for 10 min. Eventually, the resulting supernatant was collected and stored at −20 °C in the dark for the following experiments. For the assessment of the obtained extract concentration, an aliquot was freeze-dried and the dried mass weighed. For this purpose, 1 mL of the extract was frozen at −80 °C for 24 h and then freeze-dried for 12 h.

### 2.2 Characterization of the Sumac Extract

The molecular composition of the extracts was analyzed by using a Vanquish Horizon UHPLC coupled to a Q-Exactive Orbitrap mass spectrometer. The extracts were diluted (1:10 dilution) in methanol, and 5 μL of samples was directly injected into the reverse phase (RP) column. The molecular separation was carried out at 40 °C with an ACQUITY C18 BEH 1.7 μm, 2.1 mm × 100 mm column (Waters S.p.A.). The linear gradient started from 1% B phase (acetonitrile, 0.1% formic acid) and 99% A (H_2_O, 0.1% formic acid) to 100% B in 15 min with a 250 μL/min flow rate; then, the columns were stabilized for 5 min with 100% phase B. The experiments were performed in data-dependent acquisition mode alternating full MS and MS/MS scans. The precursors were ionized using an electrospray at −3.5 kV and +3.5 kV, and the inlet capillary temperature was held at 300 °C. Nitrogen sheath gas and nitrogen auxiliary gas were set at a flow rate of 30 and 10 arbitrary units (AU), respectively. Single MS survey scans were performed in the Orbitrap, recording a mass window between 70 and 1000 *m*/*z* with an automatic gain control (AGC) target of 10^6^, at a maximum injection time of 100 ms and a resolution of 35,000 at 200 *m*/*z*. Data-dependent MS/MS analysis was performed in top speed mode with a 2 s cycle-time with an isolation window of 1.2 *m*/*z* and an exclusion list for 2 s. The intensity threshold was set at 1.6 × 10^5^ using an isolation window of 1.4 Da. A 17,500 resolution, 105 AgC, and 50 ms maximum injection time were used for the MS2 scan. Raw data files were processed by Compound Discoverer 3.1 software. Briefly, raw files were aligned with an adaptive curve setting with 5 ppm mass tolerance and a 0.8 min retention time shift. Unknown compounds were detected with a 5 ppm mass tolerance, 3 signal-to-noise ratio, 30% relative intensity tolerance for isotope search, and 300,000 minimum peak intensity, and then grouped with 5 ppm mass and 0.2 min retention time tolerances. A procedural blank sample was used for background subtraction. Peak areas across all samples were subsequently normalized to the total area of the corresponding samples. Molecules were identified by using the mzCloud spectral library. Only the best match higher than 85 was considered.

The total antioxidant capacity of the sumac extract was analyzed by a total antioxidant capacity detection kit (Sigma-Aldrich) following the manufacturer’s instructions. The sumac extract (50 μL) or the Trolox solution (50 μL) at increasing concentrations (0, 80, 120, 160, 200, and 400 μM) was mixed with a Cu^2+^-containing solution (100 μL) and incubated at room temperature in the dark for 90 min. Then, the absorbance of the samples was assessed at 570 nm by using a plate reader (Victor3, PerkinElmer), and the antioxidant capacity of the sumac extract was evaluated as Trolox equivalent, according to the obtained standard curve.

Total phenolic group evaluation in the sumac extract was performed by using the Folin-Ciocalteu reagent assay (Sigma-Aldrich), which shows the total phenolic group content in a sample with respect to a standard compound (in this case, tannic acid at increasing concentrations: 0, 25, 50, 100, 150, 250, 500, and 1000 μg/mL). The assessment was performed in 24-well plates by adding 1580 μL of dH_2_O, 20 μL of extract (2.4 or 24 mg/mL) or tannic acid, 100 μL of Folin-Ciocalteu reagent, and 300 μL of sodium carbonate (20% w/v in water). After mixing, the plates were incubated at 37 °C for 35 min. Then, the absorbance of the samples was assessed at 800 nm by using the plate reader, and eventually the tannic acid-equivalent phenolic content of the sumac extract was calculated according to the obtained standard curve.

### 2.3 Preparation of Sumac Extract-Loaded Nanosheets

The nanosheets were developed through a spin coating-assisted layer-by-layer approach; the building blocks of the nanosheets, made of PCL and PDLLA, were supported by a sacrificial PVA layer. First of all, 200 μL of PCL, dissolved in acetone (5%, w/v), was spin coated on a silicon wafer surface at 4,000 rpm for 20 s. Thereafter, in order to provide a hydrophilic surface on the PCL layer, the nanosheet was plasma-treated (Ar) with a pressure of 40–60 Pa for 30 s. 140 μL of sumac extract (6 mg/mL) was thus placed on the Ar-plasma-treated PCL layer and incubated for 2 h to allow solvent evaporation. As a further step, 200 μL of PDLLA, dissolved in acetone (10% w/v), was spin coated on the extract layer at 4,000 rpm for 20 s. In order to obtain a sacrificial support layer, 200 μL of PVA in dH_2_O (10% w/v) was gently posed on the PDLLA layer and left drying at room temperature for 2 h. The nanosheets were stored at 4 °C in the dark for the following characterization and experiments. “Plain” nanosheets were obtained as controls by following an analogous procedure but skipping the deposition of the sumac extract solution.

### 2.4 Characterization of Sumac Extract-Loaded Nanosheets

Scanning electron microscopy (SEM) imaging of the nanosheets was performed with a Keyence SEM (acceleration voltage, 2 kV; magnification, 20×) after each fabrication step (i.e., after each layer deposition). Before observation, the surfaces were gold sputtered (Quorum Tech Q150RES Gold Sputter Coater) at 30 mA for 60 s.

Raman imaging was carried out with a Horiba LabRAM HR Evolution Confocal Raman Microscope equipped with a 532 nm laser. Samples were placed on Raman-grade calcium fluoride substrates (Crystran), and a 10× objective was used to acquire the signals. Signal maps were obtained according to the signal of PCL (in green, Raman shift range: 1029–1175 cm^–1^), sumac extract (in red, Raman shift range: 1728–1831 cm^–1^), and PDLLA (in blue, Raman shift range: 2872–2969 cm^–1^) with a pixel intensity proportional to peak intensity (LabSpec 6 software).

The thickness of the nanosheet layers was measured by using a stylus profilometer (DekTak, Bruker; scan range 6.5 μm). PCL, PCL-sumac, and PCL-sumac-PDLLA layers were prepared and their thicknesses separately measured. Five measures were acquired on random surface points to calculate average ± standard deviation.

Water contact angle measurements were performed on both sides (PCL and PDLLA layers) of the nanosheets by using the sessile drop method with a JC2000D contact angle analyzer (Powereach). Three measures were acquired for each sample, on a random surface point, and the results showed as average ± standard deviation.

The mechanical properties and the adhesion strength of the nanosheets were measured with a Shimadzu tensile testing equipment (EZ-S, 5 N, Shimadzu). Both extract-loaded and plain nanosheets were characterized. Samples were manually cut with the aid of a blade in 25 mm × 25 mm pieces. The static tensile tests were carried out in pulling mode at a speed of 10 mm/min. The tensile strength was considered as the highest stress, and the Young's modulus *E* was calculated according to eqs 1–3, where *F*, *h*, and *b* respectively represent the tensile load [N], the sample thickness [mm], and the sample width [mm]. *L*_0_ and *L* are the initial sample length and its length at the end of elastic behavior.
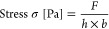
1
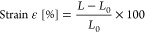
2
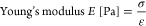
3In the adhesion test, the nanosheets were placed in contact for 30 min with an artificial skin model^16^ and thereafter peeled at a constant speed of 10 mm/min, at an angle of 90°. The force required to peel the nanosheet from the substrate was recorded as a function of the displacement. Each sample was assessed at least three times, and the results showed as average ± standard deviation. Adhesion energy was calculated according to eq 4, where *F* is the load force and *l* is the stroke at the end of the test.
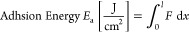
4The sumac extract cumulative release profile was indirectly evaluated by assessing the total antioxidant capacity of the eluate, compared to a standard curve obtained with increasing concentrations of the extract. Tests were carried out on a fixed size of the nanosheets (1 cm^2^) at different pH values (7.4 and 4.5), mimicking different physiological skin conditions, up to 72 h. For each time point and pH value, the eluate was processed according to the procedures previously described by using a total antioxidant capacity detection kit (Sigma-Aldrich). All experiments were performed in triplicate and results reported as average ± standard deviation

### 2.5 *In Vitro* Studies

Human dermal fibroblasts (HDFs) were cultured in Dulbecco’s modified Eagle’s medium supplemented with 10% v/v of fetal bovine serum (FBS; Gibco), 1 mM of l-glutamine (Gibco), and 100 IU/mL of penicillin–streptomycin (Gibco). The cells were incubated at 37 °C under a 5% CO_2_ atmosphere and used for experiments within 3–10 passages.

The metabolic activity of cells was assessed by using WST-1 colorimetric assay (BioVision), following the manufacturer’s instructions. All cellular experiments were performed using Transwell inserts (3 μm diameter pores; Corning) in 24-well plates, in order to have a more faithful simulation of the real application modality (i.e., nanosheets in contact with a barrier releasing drug to cells over the barrier itself). HDFs were seeded on the abluminal side of the Transwell inserts at a density of 1 × 10^4^ cells/cm^2^ and incubated for 24 h at 37 °C to promote adhesion. Subsequently, the Transwell inserts were placed in the wells containing 600 μL of medium. Samples were placed in the luminal compartment of the Transwells: 35 μL of sumac extract (corresponding to the quantity of the sumac in 1 cm^2^ of sumac extract-loaded nanosheet), plain nanosheets, or sumac extract-loaded nanosheets (1 cm^2^); the well was thereafter filled with 200 μL of medium. Cultures were incubated for 24 h at 37 °C; at the end point, the medium in the wells was replaced with WST-1 reagent (10% v/v in cell medium), and cells were incubated for a further 45 min. Eventually, absorbance was measured at 440 nm by using the microplate reader; experiments were performed in triplicate.

Cell proliferation was evaluated by following the same procedure and on the same experimental classes, by using the Quant-iT PicoGreen dsDNA assay kit (Invitrogen) following the manufacturer’s instructions. At the end point, cells were rinsed with PBS and left under 600 μL of Milli-Q water before three freezing/thawing cycles between −80 °C and room temperature, to allow complete cell lysis. After a centrifugation step to remove cellular debris (1000*g* for 15 min), the dsDNA content was evaluated by mixing 100 μL of reaction buffer, 50 μL of cell lysate, and 150 μL of PicoGreen reagent. After a 10 min incubation under shaking at room temperature, fluorescence emission (directly proportional to the dsDNA content and thus to cell number), was measured by using the microplate reader (*λ*_ex_ = 485 nm, *λ*_em_ = 535 nm); experiments were performed in triplicate.

The protective effects of sumac extract-loaded nanosheets against ROS production were tested following a *tert*-butyl hydroperoxide (tBH; Sigma-Aldrich) treatment, as a pro-oxidant insult. Control and sumac extract-loaded nanosheets experimental classes were considered, in the configuration previously described, both insulted (200 μM tBH) and non-insulted (0 μM tBH) with the pro-oxidant stimulus. After 24 h of incubation, cells were washed with PBS, collected using trypsin, and centrifuged at 1000*g* for 7 min. The cells were stained using 5 μM CellROX Green Reagent (Invitrogen) in PBS for 30 min at 37 °C and analyzed by flow cytometry (Beckman Coulter CytoFLEX; *λ*_ex_ = 498 nm, *λ*_em_ = 522 nm).

In order to evaluate the mobility of the HDFs treated with sumac extract-loaded nanosheets, a wound scratch assay was performed, aiming at the evaluation of the expansion of a cell population on a surface following an injury. Scratches were applied as linear wounds in cell monolayers by using a 200 μL sterile pipet tip; any cellular debris was removed by washing the abluminal side of the Transwell with PBS. Cells were then stained with 1 μM calcein AM (Invitrogen) and assessed at 0 and 24 h of incubation. At least three representative images from each sample were considered, and experiments were performed in triplicate. Collected data were analyzed using the ImageJ software with the “Wound Healing” plug-in (https://imagej.nih.gov/ij/plugins/index.html).

### 2.6 Statistical Analysis

Data were analyzed using analysis of variance (ANOVA) followed by Bonferroni’s *post-hoc* test in order to evaluate for significance, which was set at *p* < 0.05; data were presented as mean value ± standard deviation of three independent experiments.

## 3 Results and Discussion

### 3.1 Production and Characterization of Sumac Extract

**Figure 1:**
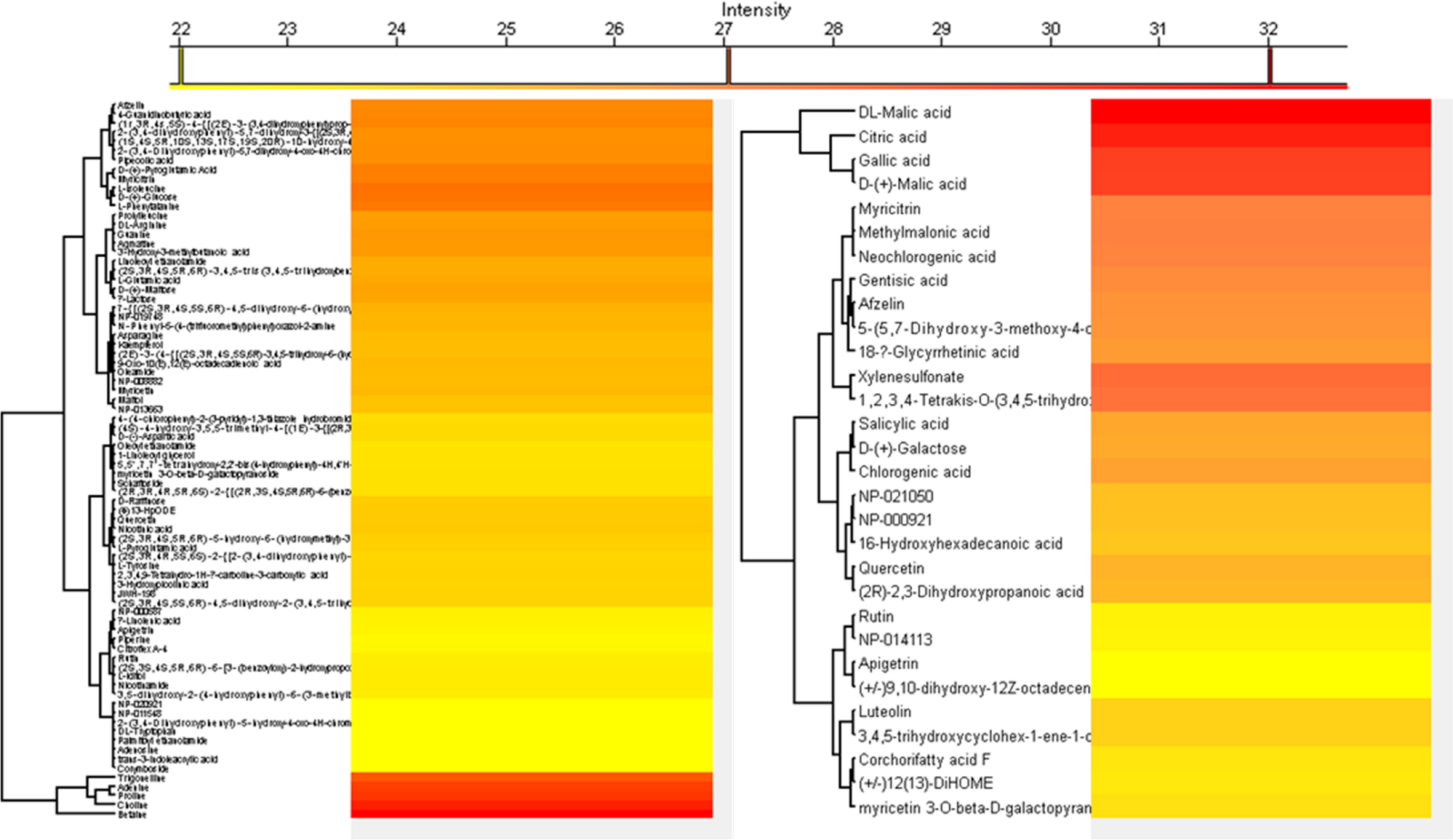
Mass spectrometry analysis of the sumac extract showing the relative amount of the compounds detected by using a C18 column in positive (on the left) and negative (on the right) ion mode.

The qualitative analysis of the sumac extract was performed by mass spectroscopy in order to highlight the extracted bioactive components, using a C18 column in positive and negative ion modes. This analysis offers also a relative quantification of the present compounds, as indicated by the heatmaps depicted in Figure 1.

Betaine, the compound present at the highest amount, acts as an intracellular osmolyte, regulating cell volume and tissue integrity. In particular, betaine plays a key role in the osmotic regulation of renal medulla cells, since they are normally exposed to high extracellular osmolarity during the urinary concentrating process.^17^ Choline, present at high content as well, is part of the cellular membrane and can be found among the membrane phospholipids; moreover, it is required in the central nervous system as a precursor of the neurotransmitter acetylcholine. Although it is considered a nonessential nutrient, observations in people deprived of dietary choline indicate the development of pathological dysfunctions, especially fatty liver disease and muscle damage,^18^ that are reversed when higher choline intake is supplied. Trigonelline is a metabolite that contributes to the stabilization of glucose and lipid levels in the blood, besides having neuroprotective and antibacterial effects.^19^ Malic acid is among the most important organic acids in wine production and plays a major role in providing microbial stability and in malolactic fermentation, a process that reduces the acidity of grapes; moreover, it gives an organoleptic character to the wine.^20^ Myricitrin, a bioactive phenolic compound, presents strong antioxidant and anti-inflammatory effects.^20^

In addition to the above-mentioned components, many other biologically active molecules, including amino acids, fatty acids, and metabolites, were also identified in the extract, all of them presenting relevant properties particularly important in biomedicine, being characterized by antibiotic, antioxidant, and anti-inflammatory features.^21,22^

The total phenolic content analysis of the extract, evaluated through the Folin-Ciocalteu test, highlighted that an equivalent of 964.4 ± 6.4 μg of tannic acidic is present in 1.2 mg of sumac extract; according to the total antioxidant capacity test, the same amount of extract has instead the antioxidant power of 92.2 ± 2.3 ng of Trolox. The high phenolic content and antioxidant capacity of the extract translate into the preserved antioxidant bioactivity of the phenolic agents;^23^ however, also several other compounds detected through mass spectroscopy (including vitamins and fatty acids) most probably contribute to the overall antioxidant activity of the sumac extract.

### 3.2 Nanosheet Characterization

The physical characteristics of each layer of the nanosheet play important roles in providing optimal wettability, tensile and adhesion strength, and extract release profile. In order to fulfill these requirements, the structure of the layers was tuned by tailoring the PCL and PDLLA molecular weights and concentrations.

**Figure 2:**
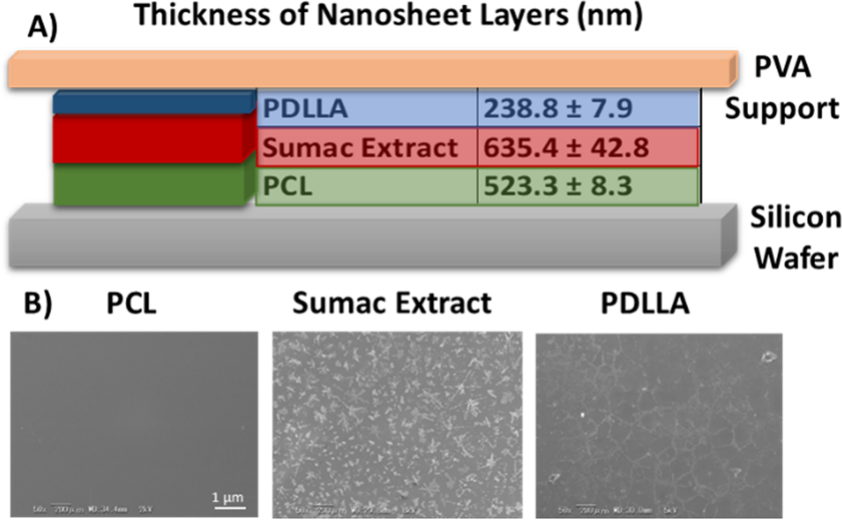
Characterization of the nanosheets. Schematic representation of nanosheet layers, with the indication of their thickness (A). Representative SEM images of the PCL, sumac extract, and PDLLA layers of the nanosheet, showing their uniform surface along with the granular deposits that occur after the sumac extract layer deposition (B).

The final nanosheet structure is depicted in the schema of Figure 2A. Over a silicon wafer, a PCL (14 kDa) solution (5% w/v) was spin coated; as a result, we obtained a polymer layer of 523.3 ± 8.3 nm. Aiming at obtaining a well-distributed sumac extract deposition above the PCL layer, plasma treatment was performed, as already anticipated, in order to mitigate the hydrophobic features of PCL. After the sumac extract was placed, a layer of 635.4 ± 42.8 nm was obtained. Eventually, we have chosen a PDLLA (15 kDa) solution (10% w/v) for the final layer, which resulted in being 238.8 ± 7.9 nm in thickness. These data were obtained from measurements of at least 10 different points of each layer: the low standard deviations of the thickness of the PCL and PDLLA layers suggest the presence of uniform surfaces, while the relative higher values assessed for the sumac extract layer thickness highlight a consistent roughness of this layer.

The morphological characterization of the nanosheet layers (PCL, sumac extract, and PDLLA) was qualitatively carried out through SEM imaging, suggesting the achievement of a uniform surface after each deposition (Figure 2B), with the sumac extract being characterized by a consistent presence of granular deposits.

**Figure 3:**
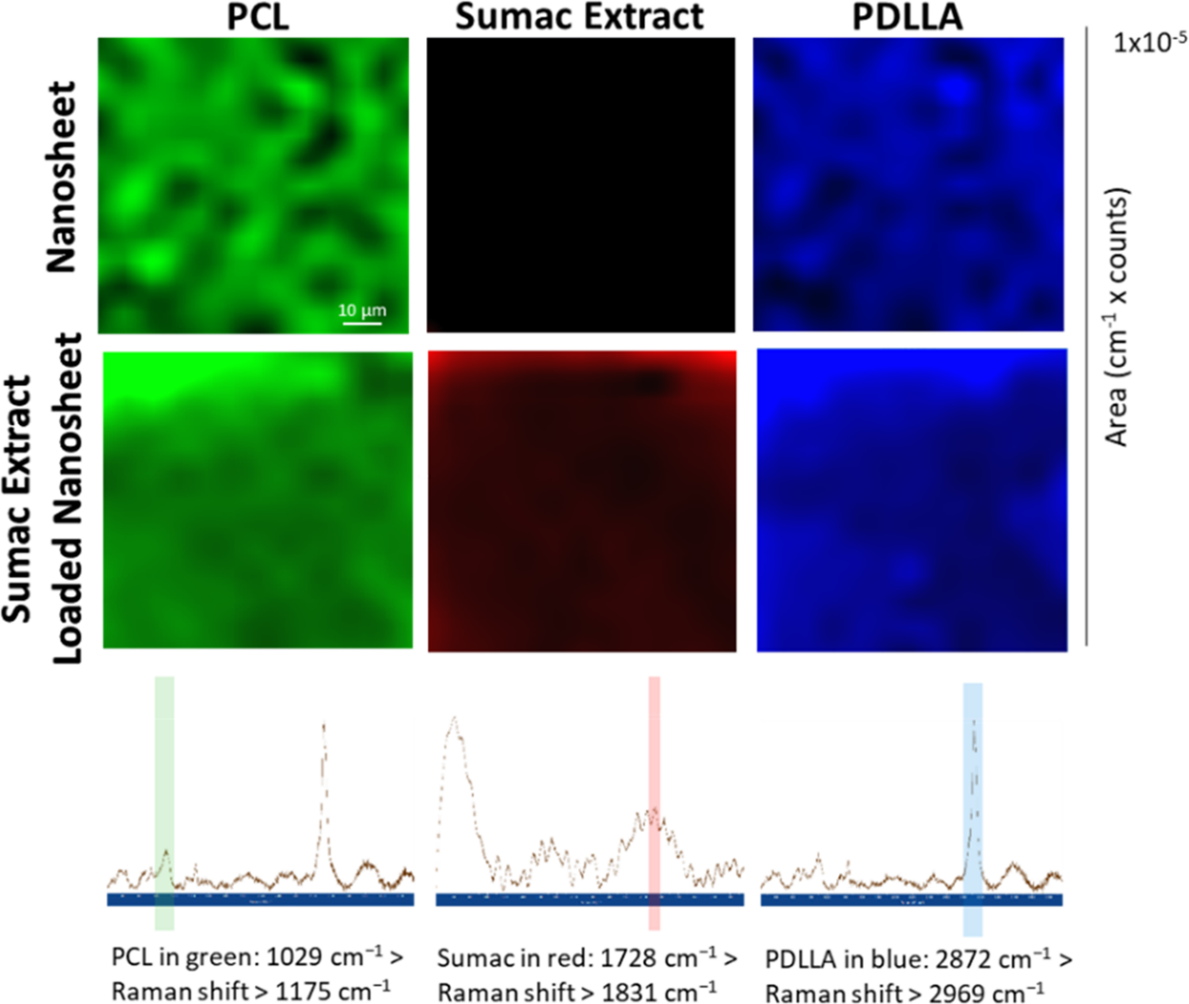
Raman images of PCL, sumac extract, and PDLLA layers of the nanosheet. Each color represents a specific peak belonging to each component (green for PCL, red for sumac, blue for PDLLA)

The different layers of the nanosheets were also analyzed through Raman spectroscopy and imaging (Figure 3), in particular by detecting fingerprints of PCL (Raman shift range: 1029–1175 cm^–1^),^24^ of sumac extract (Raman shift range: 1728–1831 cm^–1^),^25^ and of PDLLA (Raman shift range: 2872–2969 cm^–1^).^25^ More in detail, PCL has several peaks at 913 cm^–1^ (C—COO), 1003–1110 cm^–1^ (skeletal stretching), and 2800–3200 cm^–1^ (—CH), which are referred to its crystalline fraction.^24^ In sumac extract, the peak at 3337 cm^–1^ is attributed to the —OH stretching vibration, while the peak at 2940 cm^–1^ to the vibration of aliphatic hydrocarbons (—CH and —CH_2_). The peak at 1732 cm^–1^ is due to the carbonyl group (C=O), and the peaks at 1670 and 1540 cm^–1^ correspond to the aromatic ring stretching vibration. The peaks at 1248 and 1025 cm^–1^ are attributed to the ethereal C—O asymmetric stretching due to the pyran-derived ring structure of tannins.^25^ The characteristic peaks of PDLLA are at 2955 cm^–1^ (—CH) and at 1760 cm^–1^ (C=O stretching). The peaks at 1452 and 1292 cm^–1^ correspond instead to —CH_3_ and —CH, respectively.^26^

Raman observations confirm that PCL and PDLLA are uniformly spread in the nanosheet structure, and the sumac extract is successfully incorporated.

**Figure 4:**
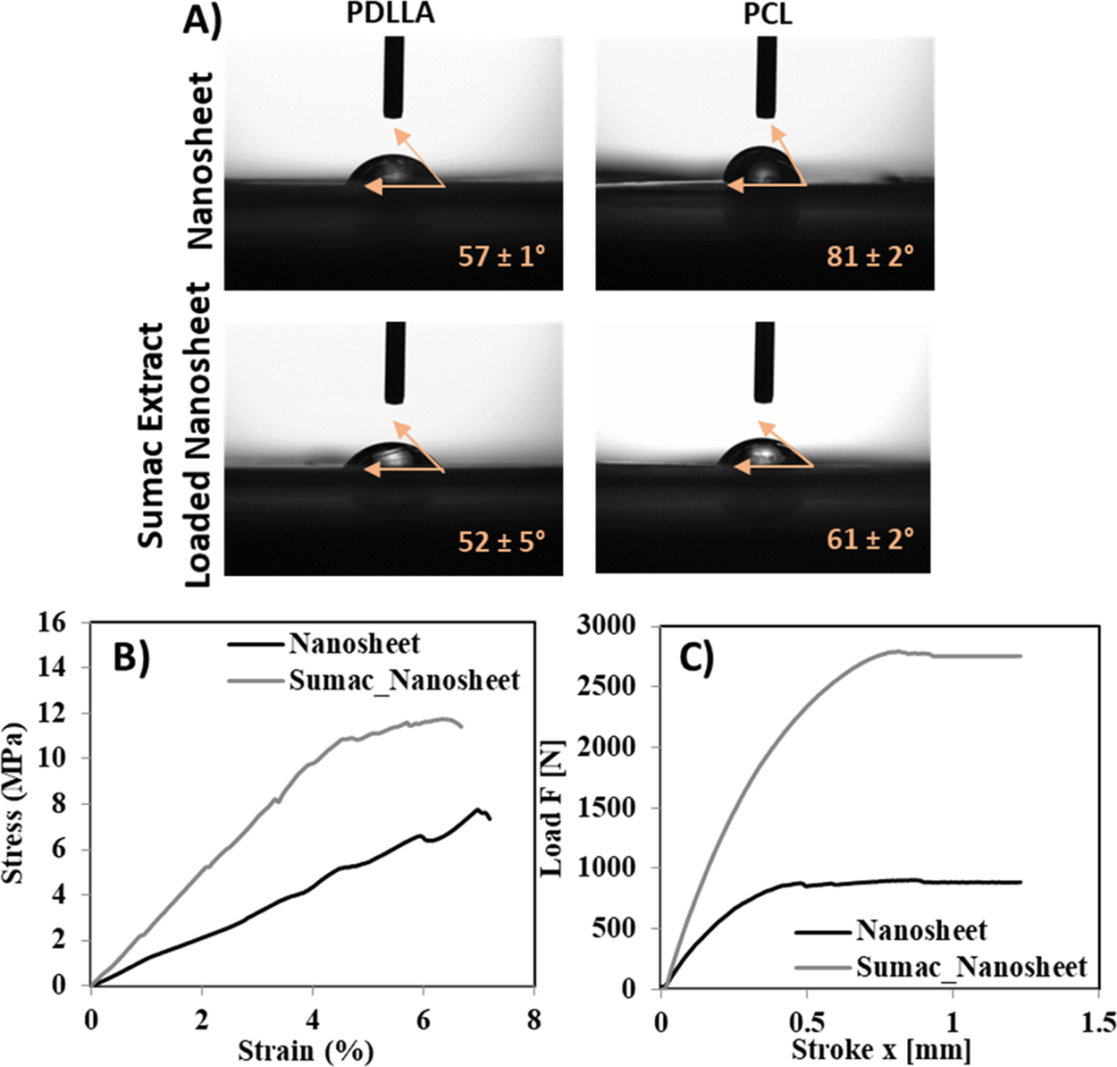
Representative contact angle measurement images of nanosheets with relative results, showing an improvement in wettability of the PCL layer due to plasma treatment (A). Tensile (B) and adhesion (C) tests on plain and extract-loaded nanosheets.

The wettability of the nanosheets was measured by contact angle assessment on both surfaces of the nanostructures (Figure 4A). The contact angle of the water just after dropping over the PDLLA layer of a plain nanosheet was found to be 57 ± 1°, while it was 81 ± 2° over the PCL layer. In the case of extract-loaded structures, a contact angle of 52 ± 5° was found for the PDDLA surface, while a contact angle of 61 ± 2° was found for the PCL ones: the effect of the Ar plasma treatment, necessary for the loading of the extract, is pretty evident by the significant reduction of the contact angle values, that highlights an improved wettability and an increased hydrophilicity ascribable to the generation of hydroxyl and carboxyl groups on the PCL surface.^25^

Regarding mechanical properties, the effects of sumac extract loading on the tensile strength and on Young’s modulus were considered. Comparing the results of the tensile test on plain and extract-loaded nanosheets (Figure 4B), we can observe that the tensile strength, i.e., the ultimate tensile strength from the initial elasticity of the stress–strain curve, was found to be 7.7 ± 2 and 11.7 ± 1 MPa, respectively. The Young’s modulus was instead calculated to be 1.1 ± 0.9 MPa for the plain nanosheets and 2.4 ± 2.2 MPa for the extract-loaded nanosheets. These data suggest a significant contribution of the extract to the increment of the mechanical properties of the nanostructures, which result in being about 1.7 times stronger and 2.0 times harder when the extract is included. One possible reason at the base of the observed data is the increment of thickness following sumac extract deposition, that is supposed to improve the tensile strength.^27^ Another possible factor is the formation of hydrogen bonds between hydroxyl groups of the phenolic compounds in the extract and the carbonyl group in PDLLA: the improved affinity between each layer is suggested to increase the mechanical strength of the whole sample as well.

Analogously, also in the evaluation of the adhesion properties, a significant difference in the behavior of plain nanosheets with respect to the extract-loaded ones was observed (Figure 4C); more specifically, when the extract is present (and thus the Ar plasma treatment has been performed), a strongly improved adhesiveness of the nanosheet on the simulated human skin is highlighted (*E*_a_ = 742.4 ± 10.5 J/cm^2^ for the plain nanosheet and *E*_a_ = 958.4 ± 10.5 J/cm^2^ for the extract-loaded nanosheets).

**Figure 5:**
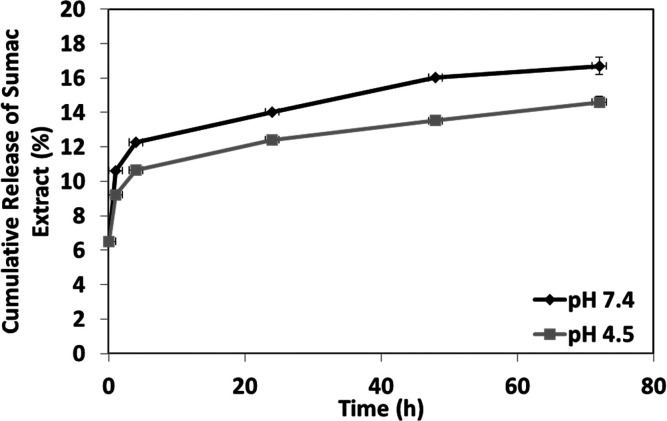
Sumac extract release from nanosheets at different pH values, up to 72 h.

Frequent high doses of drug may damage healthy cells, with consequent heavy side effects for the organism.^28^ In order to avoid such effects, a manageable and biocompatible delivery system is usually exploited, that guarantees a sustainable drug release at the required concentrations. Here, nanosheets have been considered as delivery structures especially because of their high surface area to volume ratio, that gives the advantage of exerting a therapeutic drug burst release followed by a long-lasting slow release.^29,30^ The sumac release from the nanosheet was assessed at neutral (7.4) and acidic (4.5) pH values, simulating different microenvironment conditions of intact and injured skin.^31^ Results depicted in Figure 5 highlight a well-sustained release of sumac extract from 1 cm^2^ of nanosheet up to 72 h, at both neutral and acidic pH values. More specifically, after 4 h, the sumac release was found to be 12.3 ± 0.1% at neutral pH and 10.7 ± 0.1% at acidic pH. At the end of the observation period, we found 16.7 ± 0.3% of extract released at pH 7.4 and 14.6 ± 0.5% at pH 4.5. Considering the release percentages, after 72 h, approximately 140.3 μg of sumac extract was released at pH 7.4, while 122.6 μg at pH 4.5. Despite that a high amount of extract is still contained in the nanostructures at the end of the observation window, our results support the hypothesis of a slow and well sustained drug release along the time, after a relevant burst release occurring in the first hours of tests. The slightly higher release at neutral pH could be attributed to a proven higher degradation rate of PDLLA at such pH values.^32^ This pH-dependent behavior, even if moderate, makes the system potentially suitable also in those situations where a release at neutral pH values is required. More specifically, antioxidant agent release in such an environment could be particularly interesting in the treatment of inflammatory bowel conditions.^33^

### 3.3 Biocompatibility Evaluation

**Figure 6:**
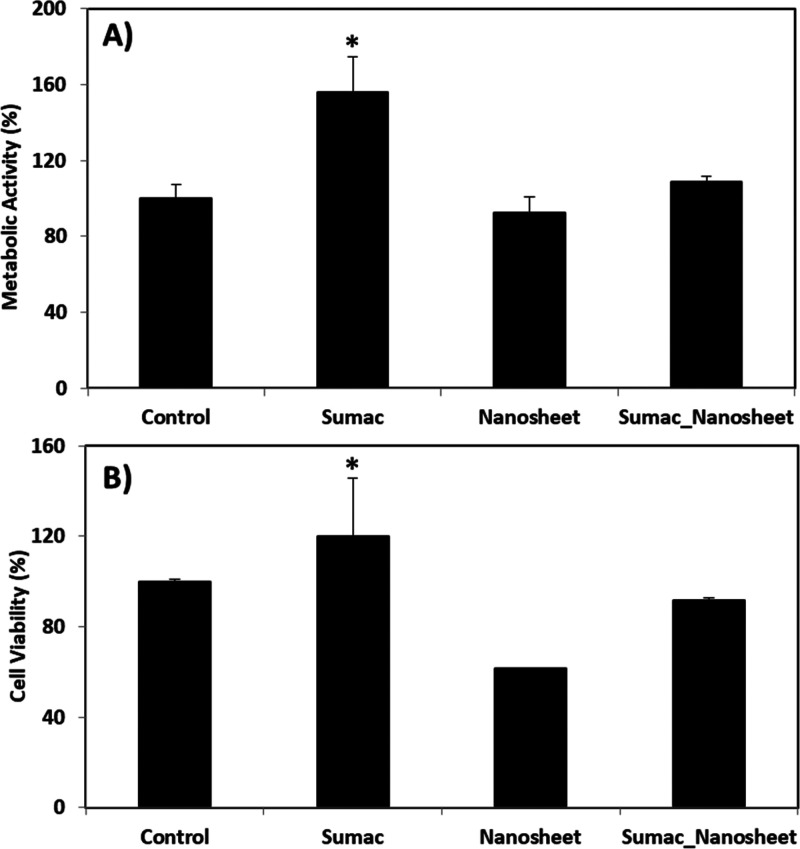
Biocompatibility evaluation of sumac extract, nanosheets, and extract-loaded nanosheets with respect to control cultures: WST-1 metabolic assay (A) and PicoGreen dsDNA quantification (B). Data are represented as mean value ± standard deviation (*p < 0.05, *n* = 3).

The biocompatibility of extract, nanosheets, and extract-loaded nanosheets was tested by using WST-1 assay for metabolic evaluation and PicoGreen dsDNA quantification for proliferation assessment, as described in the Materials and Methods. Plots reported in Figure 6 show no statistically significant differences among the experimental classes, with the exception of the extract-treated cultures, where higher metabolic activity (155.7 ± 19.0% with respect to the control, *p* < 0.05; Figure 6A) and proliferation (119.9 ± 25.9% with respect to the control, *p* < 0.05; Figure 6B) were found after 24 h of treatment.

Considering these results, we can confirm that sumac extract addition to the cell culture medium leads to the stimulation of cell metabolism and proliferation. Indeed, thanks to their strong antioxidant capacities, the extracts have a potential cell metabolism stimulatory effect by protecting the cells against the damage caused by pro-oxidant molecules.^34^ The extract in the extracellular environment reduces the oxidants, indirectly allowing an improvement in terms of cell metabolism and proliferation, in particular when a high dose is provided as a bolus; conversely, a slow release from the nanosheets is not inducing any significant effect.^35^ A good example of this condition is reported by Nair and Varalakshmi; their study^35^ shows that *Moringa oleifera* extract, having well-known antioxidant properties, presents antiproliferative effects in cancer cells below a threshold concentration (10 μg/mL), but conversely promotes cell metabolism and proliferation above this value. Analogously, in our case, we did not observe any significant change when the sumac extract was released at lower doses by the nanosheets.

### 3.4 ROS Detection and Wound Closure

**Figure 7:**
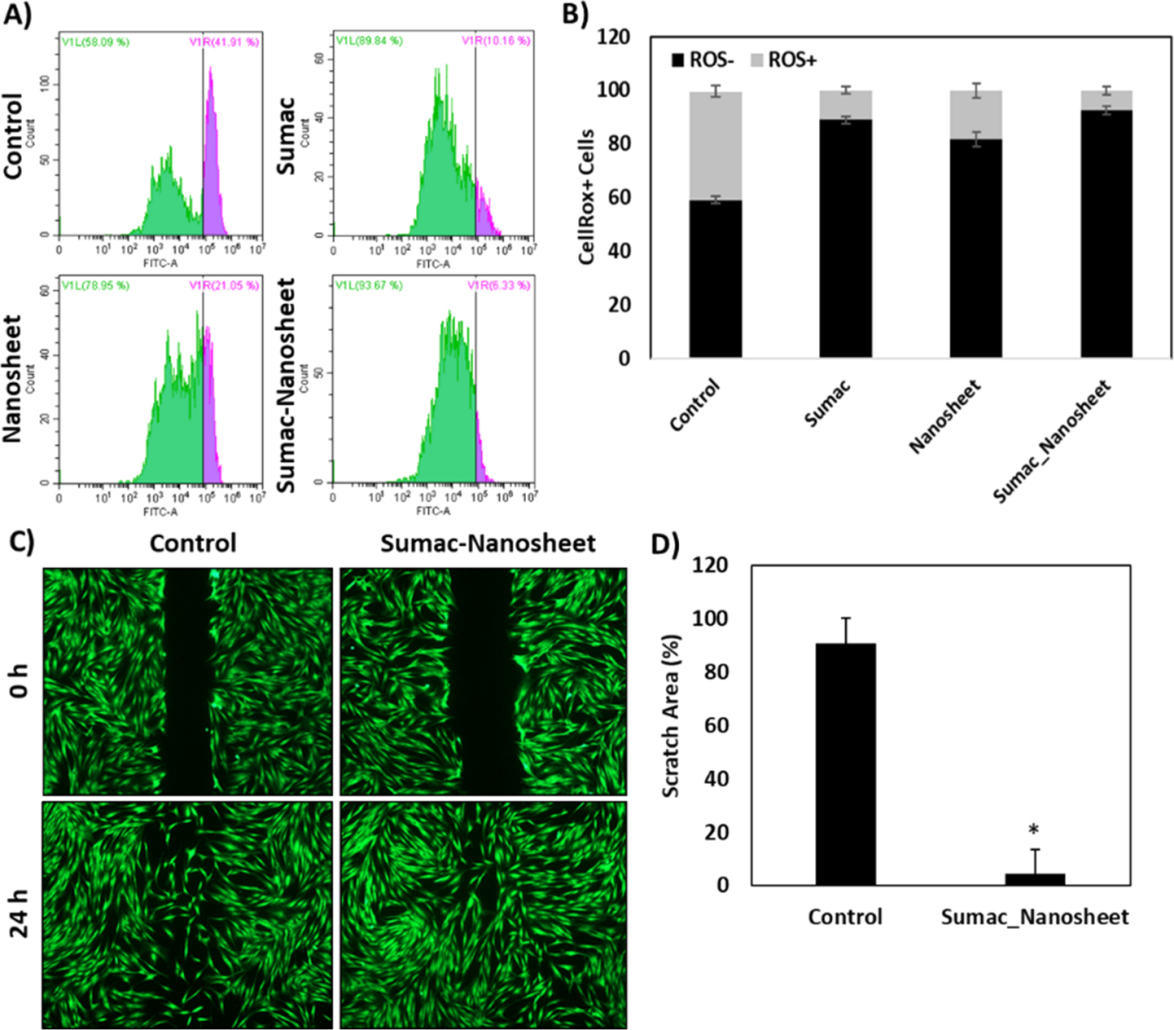
ROS evaluation through flow cytometry on HDFs after 24 h of the indicated treatments: representative scatter plots (A) and quantitative analysis (*p < 0.05, *n* = 3) (B). The obtained data demonstrate the strong antioxidant effect of the developed sumac extract-loaded nanosheets. Wound healing simulation: scratch assay. Representative fluorescent microscope images (C) and quantitative data (D). Results are expressed as the % of the initial scratch area with respect to the control (*p < 0.05, *n* = 3).

In order to evaluate the antioxidant activity of the prepared nanoplatform, experiments were performed on HDFs both under basal conditions and upon stimulation with a pro-oxidant insult, tBH. Results are summarized in Figure 7A and B, and show that following tBH treatment we observe a substantial increment of stressed cells in the cultures (40.4 ± 2.1% of ROS^+^ cells with respect to 1.4 ± 0.5% of the tBH-untreated control; data not shown), which is partially reversed by the treatment with sumac extract-loaded nanosheets (7.4 ± 1.6% of ROS^+^ cells; *p* < 0.05); ROS^+^ cells were found to be 11.1 ± 1.3% and 18.3 ± 2.7% in the cultures treated with plain sumac extract and nonloaded nanosheets, respectively.

The observed antioxidant effects are ascribable to the high content of phenolics, and in particular betaine, choline, trigonelline, and myricetin, as well as to the presence of other compounds such as vitamins and fatty acids with well-known antioxidant properties. The literature corroborates these findings: as an example, hydrolyzed tannin showed antioxidant activity against lipid peroxidation,^36^ while gallic acid plays an important role as a ROS scavenger, especially in hepatocyte cells.^37^

In the process of skin repairing, despite that ROS act as pivotal secondary messengers to enhance cell proliferation and migration, their overproduction can lead to impaired HDF function.^38^ HDFs are responsible for the generation of the connective tissues in the skin dermis and are also able to migrate through the damaged dermal tissues in order to repopulate the injured area.^39^ In this respect, a reduction of the migration capacity of HDFs might occur because of oxidative stress-dependent mitochondrial depletion, resulting in impairment during the skin regeneration process.^40^

With this all considered, we evaluated the effects of sumac extract-loaded nanosheets in a simple model of wound healing (Figure 7C and D). Considering the wound area as 100% at time 0 h (i.e., at the time of “wound” generation), the scratch area in control samples was reduced to 90.8 ± 9.4%, while that in the sumac extract-loaded nanosheet sample resulted being almost closed (4.5 ± 5.2%). The obtained results thus demonstrate that a significantly higher migration occurs in HDF cultures in contact with the sumac extract-loaded nanosheets.

The literature reports on similar conditions; for example, the wound closure effect of *Alternanthera sessilis* extract was investigated, and the migration of the cells was found to be enhanced due to the stimulation of growth factors such as vascular endothelial growth factor (VEGF), transforming growth factor beta (TGF-β), and granulocyte-macrophage colony-stimulating factor (GM-CSF).^41^ In another study, *Thymus sipyleus*, known as a therapeutic agent for skin wounds in Turkish traditional medicine, shows a cell migration stimulatory effect, ascribable to the high content of flavonoids that display a high affinity with collagen in the extracellular matrix.^42^

## 4 Conclusion

The sumac extract includes a great and diverse variety of bioactive ingredients, in particular in terms of antioxidant compounds. In order to promote their exploitation in nanomedicine by providing a controllable release, loading in a sandwich model of polymeric nanosheets was proposed. The obtained findings highlighted the successful preparation of sumac extract-loaded nanosheets and their optimal features in terms of stability, biocompatibility, and efficient ROS scavenging effects on HDFs, as well as in terms of triggered migratory stimulation. Altogether, our results encourage further investigations and applications of sumac-based nanoplatforms, with particular reference to all of those situations where a reduction of ROS overproduction is necessary.

## Data Availability

The data that support the findings of this study are available from the corresponding authors upon request.
